# Unusual RNA polymerase II patterns in meiotic prophase I in *Nannospalax xanthodon*: insights into chromatin, telomeres, and sex chromosomes

**DOI:** 10.55730/1300-0152.2794

**Published:** 2026-02-02

**Authors:** Sergey N. MATVEEVSKY, Ferhat MATUR, Faruk ÇOLAK, Andrei KANDAUROV, Mustafa SÖZEN, Svetlana V. PAVLOVA, Oxana KOLOMIETS

**Affiliations:** 1 Cytogenetics Laboratory, Vavilov Institute of General Genetics, Russian Academy of Sciences, Moscow, Russia; 2 Department of Biology, Faculty of Sciences, Dokuz Eylül University, İzmir, Turkiye; 3 Department of Biology, Faculty of Sciences, Zonguldak Bülent Ecevit University, Zonguldak, Turkiye; 4 Institute of Zoology, Ilia State University, Tbilisi, Georgia; 5 Laboratory of Population Ecology, A.N. Severtsov Institute of Ecology and Evolution, Russian Academy of Sciences, Moscow, Russia

**Keywords:** Mole rat, meiosis, synaptonemal complex, chromosome, RNA polymerase II, telomere

## Abstract

**Background:**

Meiotic transcription is a highly regulated process involving stage-specific chromatin remodeling and dynamic RNA polymerase II (RNAPII) activity. Nevertheless, our current understanding is largely based on classical model organisms, while many nonmodel mammals remain unexplored. This study investigates the transcriptional dynamics and chromatin features during meiotic prophase I in *Nannospalax xanthodon*, a subterranean blind mole rat species with extensive intraspecific karyotype variation.

**Materials and methods:**

Using immunofluorescence, we analyzed synapsis and transcriptional activity in spermatocyte nuclei of two geographically distant *N. xanthodon* males, detecting SYCP3, CREST, γH2AFX, and phosphorylated RNAPII (Ser5, Ser2). Telomere-associated proteins RAP1 and TERT were also examined to assess transcriptional activity at chromosomal ends.

**Results:**

Transcriptional activity was detected at all substages of prophase I. Unlike classical models, *N. xanthodon* exhibited no sharp pachytene-specific transcriptional reactivation; instead, RNAPII-Ser2 and Ser5 signals increased gradually from leptotene to pachytene. Notably, RNAPII-Ser5, but not Ser2, was consistently localized at telomeric regions of autosomes and sex chromosomes. These RNAPII-Ser5 foci coincided with RAP1-positive telomeres, suggesting a poised but transcriptionally inactive polymerase state at chromosome ends. The variability of RNAPII signals around the XY bivalent may also suggest a dynamic regulation of sex chromosome silencing. This is the first analysis of meiotic transcription in mole rats. The gradual transcriptional enhancement in the chromatin of prophase I nuclei and the RNAPII-Ser5 enrichment at telomeres reflect species-specific regulatory features.

**Conclusions:**

These findings highlight the importance of investigating nonmodel organisms to uncover novel mechanisms of meiotic regulation and suggest that transcriptional control during meiosis may be more evolutionarily diverse than previously recognized.

## 1. Introduction

Meiosis is a pivotal biological process essential for gametogenesis and for preserving genomic integrity across generations. During the most protracted and mechanistically complex meiotic phase, prophase I, homologous chromosomes undergo synapsis, meiotic recombination is initiated and resolved, and a coordinated transcriptional program unfolds alongside chromatin silencing events. These molecular processes collectively establish germ cell identity (Bogdanov and Kolomiets, 2007; Page et al., 2012; Turner, 2015; Zickler and Kleckner, 2023). Failure in these meiotic processes frequently triggers checkpoint responses that halt meiotic development (Subramanian and Hochwagen, 2014). Insights into transcriptional regulation at this stage have emerged from studies of RNA polymerase II (RNAPII) states, particularly analyses of its phosphorylation states at serine residues Ser5 (RNAPII-Ser5) and Ser2 (RNAPII-Ser2). These posttranslational modifications delineate distinct transcriptional phases, designating the transition from initiation to productive elongation (da Cruz et al., 2016; Harlen and Churchman, 2017; Alexander et al., 2023). RNAPII governs eukaryotic gene expression by transcribing messenger RNAs (mRNAs) and various noncoding RNAs, thereby orchestrating cellular processes such as proliferation, metabolism, and cell divisions, including meiosis. Disruption of its activity is linked to developmental disorders and diseases, underscoring its critical role in transcriptional regulation (Alexander et al., 2023; Archuleta et al., 2024).

Transcription during meiotic prophase I is subject to complex and dynamic regulation, beginning with low transcription levels (repression) during leptotene and zygotene, followed by extensive reactivation on autosomes in pachytene and diplotene (Fallahi et al., 2010; Page et al., 2012; da Cruz et al., 2016), while sex chromosomes remain transcriptionally silenced via meiotic sex chromosome inactivation (MSCI) (Turner, 2015). This program encompasses the expression of over 20,000 transcripts, many of which are essential for meiotic progression and postmeiotic differentiation (Fallahi et al., 2010; Ball et al., 2016; Fine et al., 2019). Transcriptional dynamics are closely coupled to chromatin remodeling and histone posttranslational modifications, such as H3K9 and H3K4 methylation (Wang et al., 2017), and are facilitated by transcriptional regulators that release RNAPII from promoter–proximal pausing, enabling full transcriptional progression (Alexander et al., 2023). Current insights, however, are largely derived from a limited set of model systems, underscoring the need for broader investigation.

Expanding meiotic transcriptional studies to unconventional species is crucial for identifying both conserved and species-specific regulatory mechanisms. For instance, in true bugs and lampreys, transcription resumes during zygotene independently of synapsis (Viera et al., 2017; Matveevsky et al., 2023), while marsupials exhibit distinct transcription patterns of the X chromosome and meiotic telomeres (Marín-Gual et al., 2022, 2025a). Notably, epigenetic disturbances caused by recombination defects or synapsis failures may significantly alter transcriptional programs (de la Fuente et al., 2021). Investigation of nonmodel animals with atypical meiotic characteristics or chromosomal diversity could reveal previously unknown interactions between chromatin architecture, epigenetic marks, and RNAPII dynamics. Therefore, comprehensive cross-species analysis of RNAPII and its phosphorylation states throughout prophase I is essential for deciphering the evolutionary plasticity of meiotic transcriptional programs.

In this context, we present the first detailed investigation of meiosis in Nehring’s blind mole rat, *Nannospalax xanthodon* (Nordmann, 1840), a subterranean rodent characterized by extensive chromosomal polymorphism, across Türkiye, a limited area of Georgia, and adjacent territories (Arslan et al., 2016). The study focused on chromosomal synapsis, sex chromosome behavior, meiotic telomeres, and, primarily, transcriptional activity profiling via immunodetection of phosphorylated forms of RNA polymerase II (RNAPII-Ser5 and RNAPII-Ser2) throughout prophase I, providing a qualitative overview of transcriptional patterns across meiotic stages. Our results indicate that *N. xanthodon* may exhibit distinct meiotic transcriptional patterns compared with those described in conventional mammalian models, offering new insights into the diversity of meiotic regulation in rodents. These unconventional transcriptional signatures provide novel insights into mammalian meiosis.

## 2. Materials and methods

### 2.1. Animals and ethics statement

In the present study, two adult male specimens of Nehring’s blind mole rat, *N. xanthodon*, originating from distinct localities were analyzed: one from Miasnikiani (Javakheti, Georgia), designated NX-01, and another from Kahyalar (Karabük, Türkiye), designated NX-02. The animals were intentionally sampled from geographically distant populations to corroborate specific patterns observed in meiotic analyses. The limited number of specimens was deliberately chosen in accordance with the principles of maximal conservation and minimal invasiveness, aiming to minimize the removal of individuals from natural populations while ensuring robust cytogenetic comparisons. Both individuals possessed a diploid chromosome number of 2n=50; however, they represented different cytotypes (“50-Eastern” for NX-01 and “50-Northern” for NX-02) as defined in the classification proposed by Matur et al. (2011) (see also Arslan et al., 2016). All applicable international, national, and institutional guidelines for the care and use of animals were followed. All experiments were approved by the Ethics Committee of the Vavilov Institute of General Genetics of the Russian Academy of Sciences, Russia (order no. 3 of 10 November 2016) and Zonguldak Bülent Ecevit University, Animal Experiments Local Ethics Committee (permit no. 2022-04-03/02). Mole rat specimens from Georgia were collected in accordance with Ilia State University Permit No. 7937/01 of 12 September 2018.

### 2.2. Spermatocyte microspreading technique

Spermatocyte suspensions were prepared following the methodology outlined by Kolomiets et al. (2010). The procedure for obtaining nuclear spreads was carried out according to the protocol described by Matveevsky et al. (2021) and the references cited therein. Briefly, seminiferous tissue was mechanically fragmented in phosphate-buffered saline (PBS) using fine-tipped forceps to produce a homogeneous suspension of individual cells. This suspension was subsequently mixed with 10 mM sucrose solution and incubated for 10 min. For slide preparation, aliquots of the suspension were carefully dispensed onto slides pretreated with a 1% paraformaldehyde solution prepared in bidistilled water, containing 0.15% Triton X-100. The slides were then placed in a humid chamber maintained on a level surface and allowed to fix for 2 h. After incubation, residual fixative and sucrose were removed by gently rinsing the slides in 0.04% Photoflo solution (Kodak) prepared in distilled water. Finally, the preparations were air-dried and subjected to immunofluorescence staining procedures.

### 2.3. Antibodies, immunocytochemical staining, and fluorescent microscopy

To visualize synaptonemal complexes (SCs), meiosis-specific multiprotein structures that connect homologous chromosomes and ensure their pairing, synapsis, and recombination during prophase I, we used the following primary antibodies: (1) rabbit polyclonal anti-SYCP3 (1:250; ab15093, Abcam, Cambridge, UK) and (2) mouse monoclonal anti-SYCP3 conjugated to Alexa Fluor 488 (1:250; ab2058463, Abcam, Cambridge, UK).

Centromeres were detected using (3) human anticentromere antiserum CREST (Calcinosis, Raynaud’s phenomenon, Esophageal dysmotility, Sclerodactyly, and Telangiectasia), which recognizes kinetochore proteins (1:250; #90c-cs1058, Fitzgerald Industries International, USA).

DNA double-strand breaks (DSBs) and transcriptionally silenced unsynapsed chromatin were visualized using (4) mouse monoclonal antiphospho-histone H2AX (γH2AFX; Ser139-phosphorylated H2AX) (1:1000; ab22551, Abcam, Cambridge, UK). γH2AFX is one of the most sensitive markers of DNA damage and localizes to chromatin adjacent to DSB sites.

Telomeres were analyzed using (5) rabbit polyclonal anti-RAP1 (Repressor/Activator Protein 1; TERF2IP; 1:100; NB100-56321, Novus Biologicals, USA), which detects components of the shelterin complex in meiotic chromosomes. Telomerase activity was assessed using (6) mouse monoclonal anti-TERT (1:100; MA5-16034, Invitrogen, USA), recognizing telomerase reverse transcriptase (TERT), the catalytic subunit of telomerase.

Transcriptional activity was evaluated using antibodies against RNA polymerase II (RNAPII). Specifically, (7) mouse monoclonal anti-RNAPII phosphorylated at serine 5 of the C-terminal domain (RNAPII-Ser5; 1:200; ab5408, Abcam) was used as a marker of different transcriptional states, while (8) rabbit polyclonal anti-RNAPII phosphorylated at serine 2 (RNAPII-Ser2; 1:200; ab5095, Abcam) served as a marker of active transcription.

Secondary antibodies included goat antirabbit IgG Alexa Fluor 488, antihuman IgG Alexa Fluor 546, and antimouse IgG Alexa Fluor 546/555 (Invitrogen, USA; dilution 1:300–800).

For immunofluorescence analysis, we employed both single-round and sequential double-round staining methods (Matveevsky et al., 2021). In single-round experiments, we simultaneously detected the following antibody pairs: SYCP3 with RNAPII-Ser5, SYCP3 with RNAPII-Ser2, SYCP3 with CREST, SYCP3 with γH2AFX, RNAPII-Ser2 with RNAPII-Ser5, and SYCP3 with TERT. For double-round staining, we performed sequential incubations with the following combinations: (1) SYCP3 and CREST in the first round followed by RNAPII-Ser5 in the second round; (2) SYCP3 and RNAPII-Ser5 (or RNAPII-Ser2) initially followed by CREST; (3) RAP1 and RNAPII-Ser5 first, then SYCP3; (4) RNAPII-Ser2 and RNAPII-Ser5 first, then SYCP3; and (5) RAP1 and TERT in the first round followed by SYCP3 and RNAP II in the second round.

Spermatocytes were spread onto poly-L-lysine-coated slides and subjected to a multiround, sequential immunofluorescence staining protocol to detect multiple antigens within the same cells. In the first round, slides were incubated with primary antibodies for 12 h at 4° C, followed by three washes in PBS (2–3 min each) to remove unbound antibodies. Appropriate secondary antibodies were then applied and incubated either for 4–6 h at 37 °C or for 12 h at 4 °C. After this initial staining, cells were imaged using a Zeiss Axio Imager D1 system equipped with an Axiocam HRm CCD camera, and the positions of individual cells were documented using the microscope’s coordinate grid and recorded in the laboratory log.

Following imaging, the slides underwent extensive washing in PBS (4–6 washes of 6–8 min each) to thoroughly remove residual fluorophores and prevent signal carryover. The second staining round was initiated with a distinct set of primary antibodies, followed by incubation with the corresponding secondary antibodies. Again, cells were imaged and photographed, and slides were washed thoroughly before proceeding to the subsequent round.

Successful multiround immunostaining requires careful attention to several critical parameters. First, the fluorescence of previously applied secondary antibodies must be sufficiently quenched between rounds, which was achieved by controlled exposure under a halogen lamp and extended PBS washes. The duration of interround washes is typically longer than washes conducted within a single staining round to ensure complete removal of residual fluorophores. Second, the characteristics of the target structures—including size, accessibility, and fluorescence intensity—must guide the order in which antibodies are applied. Smaller or less intensely fluorescing structures should generally be stained in earlier rounds, whereas antibodies recognizing bright, dominant signals (e.g., CREST antibodies in many species) are optimally applied in later rounds to prevent their signal from overshadowing weaker signals.

Additional considerations include the stability of fluorochromes to photobleaching: for instance, Alexa Fluor 555 conjugates were relatively resistant to quenching and therefore suited for application in second or third rounds. Careful optimization of antibody concentration and volume for each round is essential to maintain signal specificity and reproducibility. Multiround staining experiments should always be cross-validated with single-round staining to ensure that antigen distribution patterns are accurately interpreted.

Finally, after completion of all staining rounds, slides were mounted in DAPI-containing Vectashield (Vector Laboratories) and analyzed using the Zeiss Axio Imager D1 system. Images were processed and assembled using Adobe Photoshop CS5, enabling comprehensive visualization of multiple immunolabels within the same cellular context.

Immunostaining specificity for RNA polymerase II was verified using negative controls. These consisted of DAPI-only slides, slides with only primary antibodies, and slides with only secondary antibodies. The controls revealed minimal nonspecific binding and no autofluorescence.

### 2.4. Metaphase chromosomes

Suspensions of metaphase chromosomes were obtained from primary fibroblast cell cultures derived from tail biopsies of animals and established according to the standard cell culture protocol (Freshney, 2010), with modifications described earlier (Pavlova et al., 2018). Air-dried mitotic chromosome spreads were conventionally stained with 2% Giemsa for 1–2 min and sequentially subjected to differential staining. The standard trypsin/Giemsa staining technique (GTG-banding) was used to identify each chromosome arm by G-bands (Seabright 1971). CBG-banding was performed by the standard technique (Sumner 1972) to determine C-heterochromatin blocks. An ES-Experts BMR–1400HM–U CCD camera mounted on an Olympus BX43F fluorescence light microscope was used to capture images with the Argus KARIO software package (ArgusSoft, St. Petersburg, Russia). All images were processed in Adobe Photoshop 2021.

## 3. Results

### 3.1. Chromosomal synapsis and prophase I dynamics of mole rat spermatocytes

**Figure 1:**
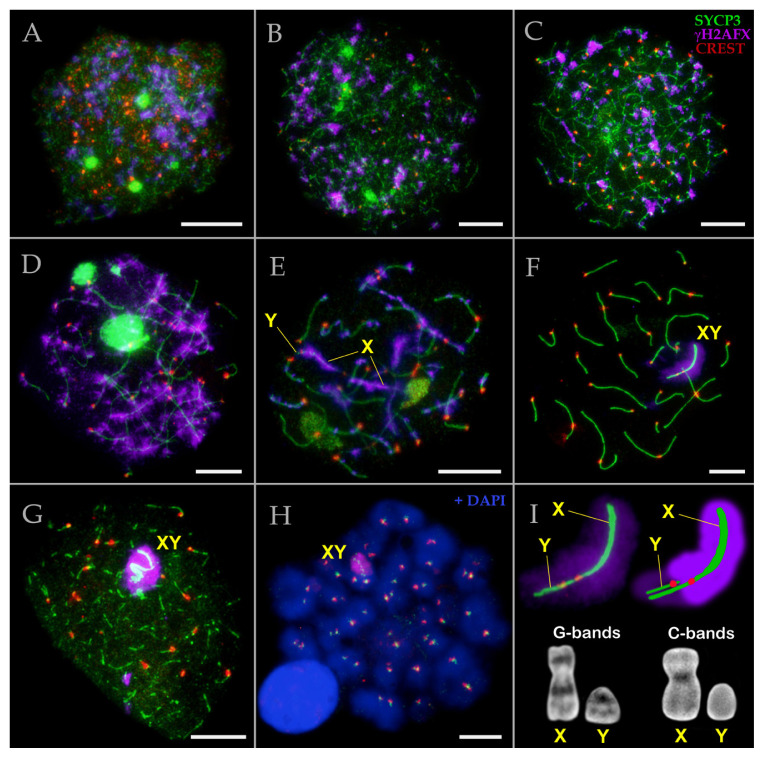
Chromosomes and meiotic dynamics of *N. xanthodon*, NX-01 (A–I). A. Early leptotene; B. Mid leptotene; C. Late leptotene–early zygotene; D. Mid zygotene; E. Late zygotene; F. Mid pachytene; G. Diplotene; H. Diakinesis; I. Sex chromosomes. Prophase I spermatocytes of mole rat stained for SYCP3 (chromosome core protein; green), for centromeres (kinetochore proteins by CREST antibody; red) and γH2AFX (violet) showing DNA double-stranded ends and inactive chromatin in sex chromosomes. DNA/chromatin was stained with DAPI (blue; for H panel only). Abbreviations: X: female sex chromosome; Y: male sex chromosome; XY: sex body. Sex bivalent, its scheme, G- and C-banded metaphase sex chromosomes are presented in the I panel. X chromosome contains C-positive heterochromatic block in centromeric region, Y chromosome is completely C-negative. Scale bar, 5 μm.

To elucidate the chronology of meiotic progression, a detailed reconstruction of chromosome synapsis and chromatin silencing dynamics was essential. Immunocytochemical detection of protein markers enabled the alignment of these processes with distinct meiotic stages. Specifically, we investigated meiotic prophase I in mole rat spermatocytes using antibodies against SYCP3, a major component of axial/lateral of SCs; CREST, a centromeric marker; and γH2AFX, a marker of DSBs and unsynapsed chromatin) (Figures 1A–1I). It is important to note that both *N. xanthodon* males from distant populations (NX-01 and NX-02) exhibited identical meiotic patterns.

In early leptotene, SYCP3 appeared as dispersed dots and short linear fragments across the whole nucleus (Figure 1A), which elongated by mid leptotene (Figure 1B). By the leptotene-to-zygotene transition, fully formed axial elements (axes) were evident (Figure 1C), consistent with the established stages of synaptic progression between homologs. Notably, γH2AFX staining exhibited a diffuse, cloud-like distribution during leptotene, though certain chromatin domains and parts of axes remained unlabeled (Figures 1A–1C).

During mid zygotene, a subset of chromosomes appeared fully synapsed, while others had just initiated synapsis (Figure 1D). At this stage, intense γH2AFX signals were detected along unsynapsed chromosomal axes (Figure 1D). By late zygotene, the majority of bivalents exhibited complete synapsis, although most retained separate γH2AFX foci, indicative of unrepaired DSBs and incomplete SCs assembly (Figure 1E). Some of the bivalents maintained asynaptic regions and remained γH2AFX-positive (Figure 1E). Sex chromosomes were discernible at this stage. The X and Y chromosomes displayed elongated axes that had only begun to synapse (Figure 1E). The X chromosome exhibited abundant γH2AFX foci along its length, excluding centromeric and pericentromeric regions, whereas the Y showed minimal γH2AFX signal (Figure 1E). Centromeric staining confirmed the acrocentric morphology of the Y, while the X appeared to be submetacentric, as supported by G- and C-banded metaphase chromosomes (Figure 1I). Notably, meiotic chromosomes in nuclei remained tethered to the nuclear envelope. Prior to full synapsis, uneven stretching of chromosomes occasionally obscured precise morphological classification (e.g., distinguishing submetacentric from metacentric types).

During mid pachytene, SCs assembly was completed on all autosomes (Figure 1F). The XY pair was readily identifiable in most pachytene nuclei due to its distinctive configuration: typically presenting as a sex bivalent with nonaligned centromeres, featuring complete Y chromosome alignment to the X (lacking any unpaired Y segment) and a thickened unsynapsed region of the X (Figure 1F and 1I). The sex chromosome morphology was more clearly discernible at this stage, as confirmed by G- and C-banded metaphase chromosome analysis (Figure 1I). DAPI staining revealed that most mid pachytene nuclei lacked a distinct sex body domain. The entire XY bivalent was enveloped in a γH2AFX cloud, with slightly more intense and condensed signal surrounding the asynaptic portion of the X (Figures 1F and 1I).

In diplotene, bivalents underwent desynapsis, with SCs appearing fragmented (Figure 1G). The sex bivalent adopted a compact configuration enveloped by γH2AFX signal (Figure 1G). This observation suggests that during late pachytene, the XY body may form a distinct DAPI-dense domain at the nuclear periphery, as further corroborated by additional micrographs presented below. At diakinesis, SYCP3 signal persisted exclusively at centromeric regions, while sex chromosomes exhibited punctate SYCP3 staining and remained surrounded by a small γH2AFX cloud (Figure 1H).

### 3.2. RNA polymerase II distribution in mole rat prophase I spermatocytes

**Figure 2:**
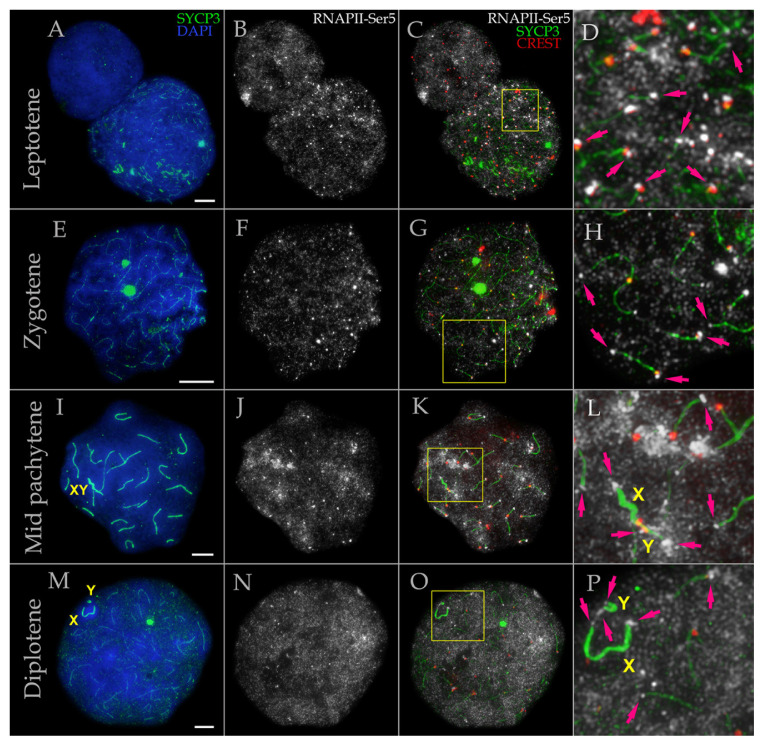
RNA polymerase II (RNAPII-Ser5) distribution in spermatocytes of *N. xanthodon* at different prophase I substages, NX-01 (A–P). A–D. Leptotene; E–H. Zygotene; I–L. Mid pachytene; M–P. Diplotene. Prophase I spermatocytes of mole rat stained for SYCP3 (chromosome core protein; green), for centromeres (kinetochore proteins by CREST antibody; red), and RNAPII-Ser5 (white) showing transcriptional chromatin regions. DNA/chromatin was stained with DAPI (blue). Magenta arrows point to RNAPII signals in telomeres of meiotic chromosomes. Abbreviations: X: female sex chromosome; Y: male sex chromosome; XY: sex body. Yellow boxes indicate the enlarged region of nuclei shown in the panels on the right (C in D, G in H, K in L, O in P). Scale bar, 5 μm.

**Figure 3:**
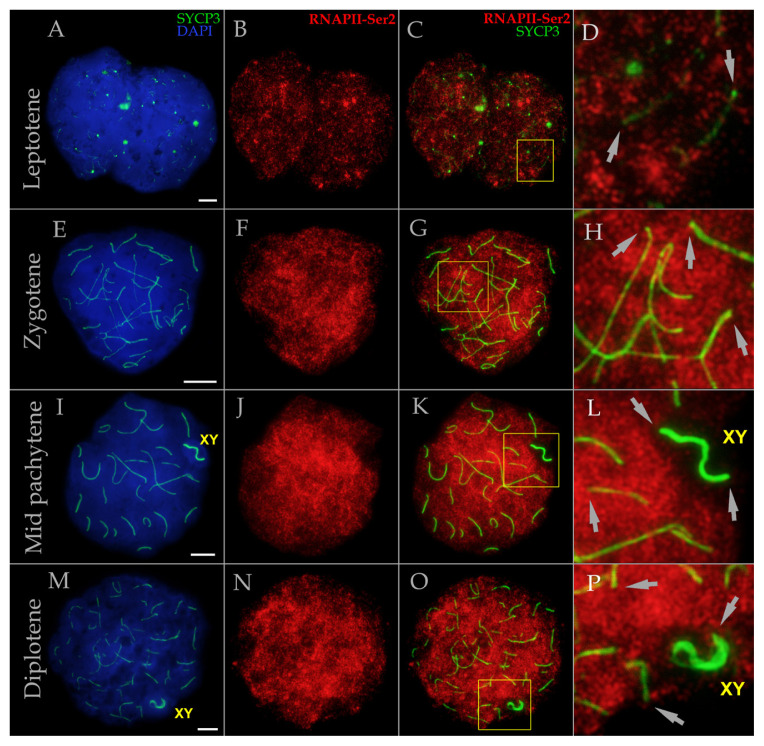
RNA polymerase II (RNAPII-Ser2) distribution in spermatocytes of *N. xanthodon* at different prophase I substages, NX-02 (A–P). A–D. Leptotene; E–H. Zygotene; I–L. Mid pachytene; M–P. Diplotene. Prophase I spermatocytes of mole rat stained for SYCP3 (chromosome core protein; green) and RNAPII-Ser2 (red), showing transcriptionally active sites. DNA/chromatin was stained with DAPI (blue). Gray arrows indicate absence of strong RNAPII-Ser2 foci in telomere regions, as observed for RNAPII-Ser5. Abbreviations: X: female sex chromosome; Y: male sex chromosome; XY: sex body. Yellow boxes indicate the enlarged region of nuclei shown in the panels on the right (C in D, G in H, K in L, O in P). Scale bar, 5 μm.

To investigate transcriptional activity within the prophase I nucleus, we studied the distribution of phosphorylated RNA polymerase II (RNAPII-Ser5 and RNAPII-Ser2) (Figures 2A–2P, 3A–3P, and S1). Our analysis revealed that during both leptotene (Figures 2A–2D) and zygotene (Figures 2E–2H), nuclei exhibited intense RNAPII-Ser5 signal manifested as numerous dot-like foci of varying sizes, independent of chromosomal synapsis. The RNAPII-Ser5 signal was moderately more intense in pachytene-stage nuclei, appearing as brighter foci with substantially decreased spacing between them, suggesting a denser distribution (Figures 2I–2L). Notably, certain nuclear regions contained more pronounced RNAPII-Ser5 domains that were preferentially associated with chromatin near the terminal regions of some bivalents. During diplotene, nuclei maintained uniformly distributed, intense RNAPII-Ser5 signal without cloud-like accumulations (Figures 2M–2P). At this stage, a clearly formed sex body was visible at the periphery of the meiotic nucleus (Figures 2M–2P).

During leptotene, the RNAPII-Ser2 signal appeared as numerous foci and globular aggregates (Figures 3A–3D). In some leptotene cells, the RNAPII-Ser2 signal appeared slightly stronger (Figure 3A–3D), whereas in others it was weaker (compare Figures S2 and S3), likely reflecting dynamic changes in signal localization and intensity across leptotene substages. At the zygotene stage, all spermatocytes displayed a more pronounced RNAPII-Ser2 signal compared with leptotene (Figures 3E–3H, S3, S4). In pachytene (Figures 3I–3L, S2, S4) and diplotene (Figures 3M–3P), the RNAPII-Ser2 signal was highly intense, with minimal interfocal distances, indicating an exceptionally dense distribution pattern. Although the overall intensity of RNAPII-Ser2 was comparable between mid zygotene and mid pachytene, the distance between individual RNAPII-Ser2 foci was noticeably variable, reflecting differences in their spatial organization.

**Figure 4:**
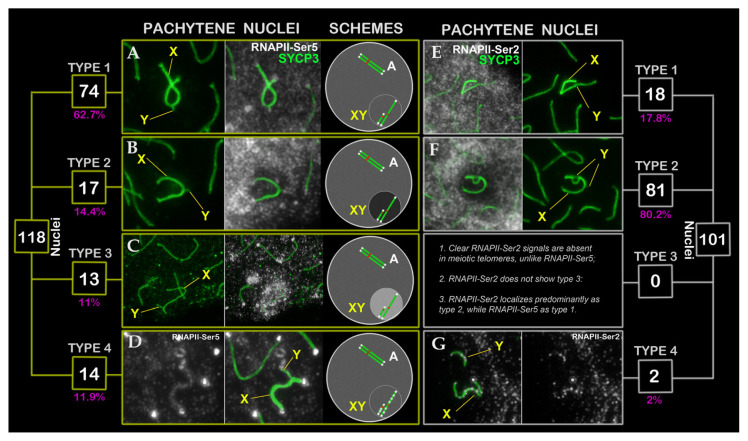
Localization variability of RNA polymerase II, RNAPII-Ser5 (left panels A–D, yellow boxes) and RNAPII-Ser2 (right panels E–G, white boxes), within the XY bivalent in mole rat (NX-02) pachytene spermatocytes. Pachytene nuclei stained for SYCP3 (green), RNAPII-Ser5 (white), and RNAPII-Ser2 (white). Four distinct patterns were observed: (1) Type 1. RNAPII-Ser5 (A) and RNAPII-Ser2 (E) signals around the XY bivalent comparable to the rest of the chromatin; (2) Type 2. Reduced RNAPII-Ser5 (B) and RNAPII-Ser2 (F) signals near the XY bivalent; (3) Type 3. Elevated RNAPII-Ser5 (C) signals specifically on the sex bivalent chromatin; and (4) Type 4. Distinct RNAPII-Ser5 (D) and RNAPII-Ser2 (G) signals along the X and Y axes. Small yellow and white squares display total cell counts (white) and their percentage (magenta). RNAPII-Ser5 signal intensity in meiotic nuclei schematics (right column) is denoted by grayscale gradients (dark to light). Abbreviations: A: autosome, X: female sex chromosome; Y: male sex chromosome; XY: sex body. While RNAPII-Ser5 localized distinctly at meiotic telomeres, RNAPII-Ser2 was undetectable at telomeres of chromosomes.

**Figure 5:**
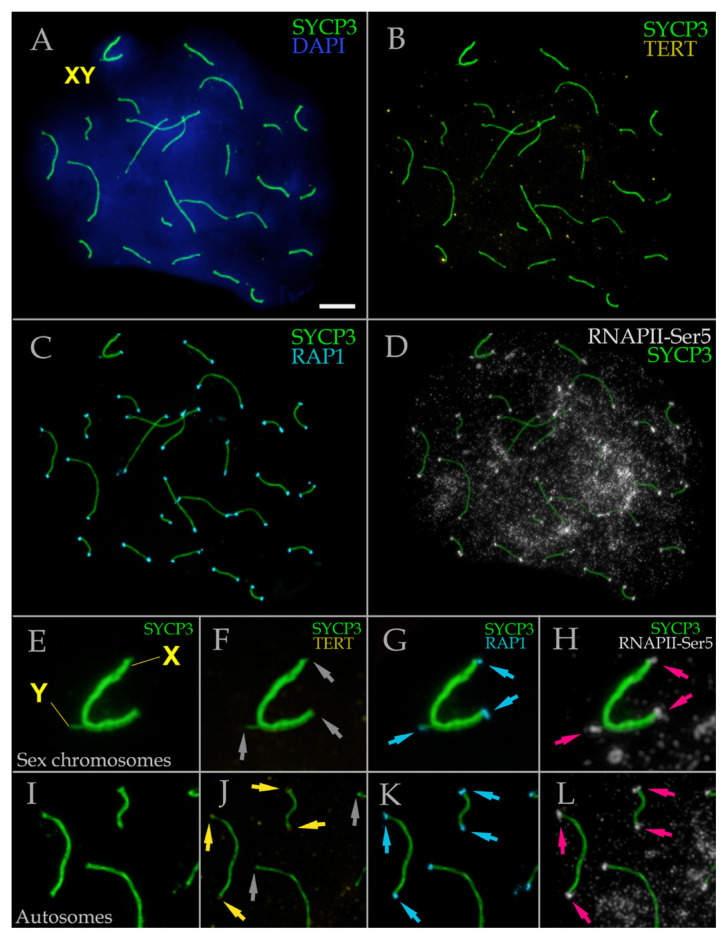
RNA polymerase II (RNAPII-Ser5) positive meiotic telomeres in pachytene spermatocyte of *N. xanthodon*, NX-02 (A–L). Spermatocyte (A–L) of mole rat stained for SYCP3 (chromosome core protein; green; A–L panels), TERT (catalytic subunit of telomerase; yellow; B, F, J panels), RAP1 (telomeric shelterin complex; cyan; C, G, K panels) and RNA polymerase II (RNAPII-Ser5; white; D, H, L panels) showing RNAPII-Ser5 positive telomeric sites. DNA/chromatin was stained with DAPI (blue; for A panel only). Yellow arrows denote weak TERT signals, grey arrows indicate TERT foci absence, cyan arrows mark RAP1 foci, and magenta arrows mark RNAPII-Ser5 signals at meiotic telomeres. Abbreviations: X: female sex chromosome; Y: male sex chromosome; XY: sex body. Scale bar, 5 μm.

Unexpectedly, we observed distinct patterns of RNAPII-Ser5 localization within the sex bivalent (Figure 4). This analysis was based on 118 pachytene nuclei of mole rat NX-02. In the majority of pachytene nuclei (62.7%, type 1), the RNAPII-Ser5 signal intensity in chromatin surrounding the XY bivalent was comparable to that in the remaining nuclear chromatin (Figures 2K and 4). In some nuclei (14.4%, type 2), however, the RNAPII-Ser5 signal was decreased around the XY bivalent compared with the rest of the nucleus (Figures 4 and 5), whereas in others (11%, type 3), an increased RNAPII-Ser5 signal was clearly localized to the sex bivalent chromatin (Figure 4). In some nuclei (11.9%, type 4), clear RNAPII-Ser5 signals were visualized along the X and Y axes (Figure 4). The RNAPII-Ser2 signal exhibited localization patterns corresponding to types 1, 2, and 4 but with a strikingly different ratio (17.8%, 80.2%, and 2%, respectively; n = 101 pachytene nuclei in mole rat NX-02) (Figures 4E–4G). When the sex bivalent formed a well-defined, separate DAPI domain (typically observed in late pachytene), the RNAPII-Ser2 signal was reduced to varying degrees compared with the rest of the meiotic nucleus (Figures 3, S1, S2, S4).

Strikingly, prominent RNAPII-Ser5 foci were consistently detected at terminal chromosomal segments, including the XY bivalent, throughout all substages of prophase I (Figure 2). This pattern was observed in both male mole rats examined. We conducted further characterization of these chromosomal termini (see Section 3.3 below).

### 3.3. RNA polymerase II and telomeric markers in mole rat spermatocytes

To characterize chromosomal ends, we performed immunodetection of the TERT telomerase subunit, which is proposed to primarily serve structural functions in meiotic telomeres (Reig-Viader et al., 2014), and of the RAP1 protein, one of six components of the shelterin complex (Li et al., 2000) (Figures 5A–5L). In mole rat spermatocytes, the TERT signal was weak (Figure 5B). Some terminal regions of SCs exhibited faint TERT foci (occasionally diffuse), with signal intensity above background but below that of certain nonspecific dots (Figures 5I and 5J). Several bivalents lacked detectable TERT signal (Figures 5I and 5J). While this could reflect technical limitations (potentially suboptimal antibody affinity for the mole rat TERT epitope), previous reports similarly observed incomplete TERT labeling in SCs (Reig-Viader et al., 2014).

RAP1 immunostaining was detected at all termini of SCs and the XY bivalent (Figure 5C). Three distinct RAP1 foci were identified in the sex bivalent: one corresponding to the telomere of the unsynapsed part of the Y, a second marking the telomere of the unsynapsed X region, and a third representing the synapsed telomeres of X and Y (Figures 5C and 5G). Coimmunodetection of RAP1 and RNAPII-Ser5 revealed similarity in size and morphology of signals between these proteins, suggesting potential colocalization (compare Figures 5C with 5D; Figures 5E–5L). Although we did not quantitatively assess colocalization using additional methods, our findings clearly demonstrate that RAP1-positive telomeres in mole rat spermatocytes consistently coincide with RNAPII-Ser5-positive telomeric regions. No distinct RNAPII-Ser2 foci were detected in the telomeres (Figures 3, S1–S4).

## 4. Discussion

Understanding chromatin dynamics during meiotic prophase I is a central challenge in modern cell and developmental biology. This phase involves extensive three-dimensional chromatin reorganization, characterized by the dissolution of topologically associating domains (TADs), a hallmark of interphase chromosomes, and the formation of large chromatin loop arrays in pachytene (Patel et al., 2019), integrated with sophisticated gene expression programs and epigenetic reorganization (de la Fuente et al., 2021; Fallahi et al., 2010; Wang et al., 2017; Fine et al., 2019). In parallel, a distinctive nuclear architecture is established (Berrios, 2017), shaped by the spatial arrangement of telomeric and centromeric domains, their interactions with the nuclear envelope, and the progression of chromosome synapsis and desynapsis, against a backdrop of differential transcriptional programs.

RNA polymerase II (RNAPII) is the central enzyme driving eukaryotic transcription, synthesizing not only protein-coding mRNAs but also a diverse array of noncoding RNAs (Archuleta et al., 2024). These include regulatory small nuclear RNAs, ribosomal processing small nucleolar RNAs, gene-silencing microRNAs, and certain small interfering RNAs (Eick and Geyer, 2013). During meiotic prophase I, the C-terminal domain (CTD) of RNAPII functions as a dynamic regulatory platform, with phosphorylation at serine residues Ser5 and Ser2 generating distinct interaction interfaces that correspond to specific transcriptional stages. Ser5 phosphorylation (RNAPII-Ser5) promotes recruitment of transcription initiation factors. It is associated with promoter-proximal pausing, whereas Ser2 phosphorylation (RNAPII-Ser2) facilitates the binding of elongation factors, supporting processive (active) transcription (Eick and Geyer, 2013; Bowman and Kelly, 2014; Alexander et al., 2023; Harlen and Churchman, 2017).

Crucially, RNAPII phosphorylation at Ser5 alone is not sufficient to infer active transcription. Extensive genome-wide and mechanistic studies have demonstrated that RNAPII-Ser5 frequently marks polymerases that have initiated transcription but remain stalled or paused near promoters or regulatory regions, without transitioning into productive elongation (Ferrai et al., 2017; Harlen and Churchman, 2017). In contrast, phosphorylation at Ser2 is widely accepted as a defining hallmark of transcriptional elongation across gene bodies. Therefore, the presence of RNAPII-Ser5 in the absence of RNAPII-Ser2 should be interpreted as evidence of transcriptional poising or promoter-proximal pausing rather than ongoing RNA synthesis (Eick and Geyer, 2013; Bowman and Kelly, 2014; Alexander et al., 2023; Harlen and Churchman, 2017). The coordinated action of these phosphorylation events enables the cell to transition between phases of transcriptional repression and activation, a process crucial for the successful completion of meiosis. These modifications convert the CTD into a signaling hub that orchestrates transcriptional progression across the meiotic genome.

This study represents the first comprehensive meiotic analysis in *N. xanthodon*, incorporating detailed sex chromosome characterization and provides the first integrated assessment of sex chromosome structure and behavior in mole rats, including G- and C-banding, chromosome synapsis throughout prophase I, DNA DSB dynamics, spatial positioning of the sex bivalent and sex body formation, meiotic silencing of sex chromatin, and RAP1- and RNAPII-defined telomeric sites. Chromosomal synapsis in *N. xanthodon* spermatocytes exhibits patterns similar to those previously described in closely related species, *N. ehrenbergi* (Matveevsky et al., 2018) and *N. leucodon* (Matveevsky et al., 2020). Intriguingly, RNAPII-Ser5 and RNAPII-Ser2 immunolocalization revealed consistent transcriptional signatures across geographically isolated cytotypes, suggesting that these regulatory mechanisms are evolutionarily conserved within *N. xanthodon* populations.

One of the most notable differences between *N. xanthodon* and classical mammalian models, such as the mouse, is observed in the regulation of transcription during meiotic prophase I. In mice, this process typically involves a phase of general repression in leptotene and zygotene, followed by robust transcriptional reactivation during the pachytene stage (Fallahi et al., 2010; Page et al., 2012; Alexander et al., 2023). In contrast, *N. xanthodon* exhibits a different pattern: transcription is attenuated in leptotene but does not completely disappear. Instead, it intensifies gradually, reaching an active state by midzygotene. This indicates that a distinct “global expression switch” or sharp transition to active transcription (Fallahi et al., 2010; Alexander et al., 2023) is absent in mole rats. Instead, transcriptional activity shows a smooth, continuous increase. Both RNAPII-Ser5 and RNAPII-Ser2 are localized within chromatin across all prophase I stages, with their distribution patterns suggesting differential transcriptional state. For example, RNAPII-Ser2 signal intensity in mid zygotene and mid pachytene was broadly comparable, consistent with a gradual rather than abrupt increase in transcriptional output. This continuous presence of both active (Ser2) and initiation/paused (Ser5) forms suggests a coordinated mechanism regulating RNAPII activity throughout prophase I.

A species-specific feature of *N. xanthodon* is the consistent presence of prominent RNAPII-Ser5 foci at chromosomal termini, including those of the XY bivalent, across all prophase I substages. These foci colocalize with RAP1-positive telomeres, suggesting a specialized role for RNAPII-Ser5, as RNAPII-Ser2 was not detected in these regions. This specific pattern in *N. xanthodon* shows partial similarity to that observed in some marsupials, such as Dasyuridae, which also display distinct RNAPII-Ser5 localization on the X chromosome and at meiotic telomeres (Marín-Gual et al., 2022, 2025a, 2025b). While these studies have interpreted RNAPII-Ser5 enrichment as indicative of transcriptional activity, it is important to note that RNAPII-Ser5 phosphorylation more commonly reflects promoter-proximal pausing or initiation stages rather than productive elongation. Consequently, the assumption that RNAPII-Ser5 signifies fully active transcription in these chromosomal regions should be regarded with caution, as it may reflect a degree of overinterpretation. The absence of RNAPII-Ser2 further supports the idea that RNAPII-Ser5 presence alone does not definitively indicate active transcription, and alternative roles such as chromatin regulation, telomere protection, or transcriptional poising remain plausible.

Several hypotheses can be proposed to explain the prominent RNAPII-Ser5 foci at mole rat telomeres, in the absence of corresponding RNAPII-Ser2 signals. First, while RNAPII-Ser5’s presence at meiotic telomeres and sex bivalents aligns with its established role in transcription initiation (Alexander et al., 2023), the absence of RNAPII-Ser2 reveals an unusual regulatory scenario. In typical somatic cells, RNAPII phosphorylated at Ser5 initiates transcription and often pauses near promoters, after which it transitions into productive elongation accompanied by Ser2 phosphorylation (Eick and Geyer, 2013; Harlen and Churchman, 2017). The persistent RNAPII-Ser5-only state in meiotic telomeres observed here suggests a meiosis-specific mechanism for maintaining transcriptionally poised but elongation-blocked polymerase, potentially safeguarding chromosomal integrity during meiotic prophase while keeping telomeric regions transcriptionally silent despite polymerase presence.

Second, given that meiotic silencing, particularly MSCI, is essential for suppressing transcription in unsynapsed chromatin (Page et al., 2012), the detection of RNAPII-Ser5 on the sex bivalents and their axes suggests its potential role in either establishing or modulating this silencing process. The observed variability in RNAPII-Ser5 localization (distributed across XY chromatin or concentrated along sex chromosome axes) may reflect dynamic chromatin restructuring during meiotic silencing. Critically, the scarcity of RNAPII-Ser2 foci in these RNAPII-Ser5-enriched regions further indicates transcriptional suppression or highly restricted, regulated activity. Together, these findings imply a coordinated regulatory mechanism where RNAPII-Ser5 helps maintain a paused or inactive polymerase state, enabling a switch between active and passive transcriptional modes in synchrony with meiotic silencing demands.

A third explanation for the prominent RNAPII-Ser5 foci at meiotic telomeres is their involvement in the transcription of telomeric repeat-containing RNA (TERRA), a long noncoding RNA transcribed by RNAPII from subtelomeric and telomeric regions (Azzalin et al., 2007; Schoeftner & Blasco, 2008; Porro et al., 2014). Recent immunocytological evidence confirms the presence of TERRA at meiotic telomeres in spermatocytes, supporting the notion of telomeric transcription during meiosis (Biswas et al., 2023). We propose that the RNAPII-Ser5 signal observed at mole rat telomeres may reflect transcriptional initiation or preinitiation complex formation associated with TERRA synthesis. The consistent absence of RNAPII-Ser2 at these sites suggests that elongation may be brief, tightly regulated, or repressed. This could indicate noncanonical transcription dynamics at telomeres, possibly involving abortive or transient elongation phases. Moreover, the role of chromatin-associated factors such as cohesin SMC1β in repressing TERRA levels through chromatin compaction (Biswas et al., 2023) underscores the complex regulation at meiotic telomeres. Thus, RNAPII-Ser5 may serve as a marker of regulated TERRA transcription, which could be associated with telomere integrity or nuclear architecture. Interestingly, TERT immunostaining in mole rat spermatocytes was generally weak and not uniformly present at all synaptonemal complex ends, with certain bivalents showing no detectable signal. While this could be due to methodological constraints, it may also indicate that TERT plays a more limited structural role in meiotic telomeres, in agreement with the possibility of nonstandard telomeric transcriptional control in *N. xanthodon*.

Together, these three hypotheses highlight the complexity of RNAPII-mediated transcriptional regulation at telomeres and sex chromosomes during meiosis and reveal potential species-specific mechanisms that warrant further investigation. Our findings demonstrate that both RNAPII-Ser5 and RNAPII-Ser2 can serve as appropriate markers for assessing transcriptional dynamics within prophase I chromatin, including potential transcriptional reactivation. Nonetheless, the distinct RNAPII-Ser5-associated immunosignatures observed in telomeres and sex bivalents warrant cautious interpretation, as they are unlikely to represent sites of active transcription per se but instead reflect intricate regulatory transitions within meiotic chromatin.

In conclusion, this work presents the first comprehensive characterization of meiotic progression and associated transcriptional dynamics in the Nehring’s blind mole rats, offering what appears to be one of the first in-depth analyses of meiotic transcription in wild animal populations. Distinct transcriptional patterns were identified and appear conserved across geographically distant populations. Notably, *N. xanthodon* lacks the sharp transcriptional reactivation typically observed in pachytene-stage spermatocytes of some mammals, instead showing a gradual increase in activity from leptotene to mid zygotene. The sustained presence of RNAPII-Ser5 at meiotic telomeres, in the absence of RNAPII-Ser2 and alongside RAP1 colocalization, points to a species-specific regulatory mechanism. Collectively, these findings underscore the evolutionary flexibility of meiotic transcriptional programs and highlight the importance of studying nonmodel species to reveal both conserved and lineage-specific regulatory strategies. Future research should aim to elucidate the molecular mechanisms underlying RNAPII-Ser5 function at telomeres and its role in telomere integrity and meiotic progression in mole rats and other animals. The conservation of these transcriptional features across two distinct cytotypes of Nehring’s blind mole rat further highlights their functional significance and raises intriguing questions about potential differences in meiotic regulation within other *Nannospalax* species, such as *N. leucodon* and *N. ehrenbergi*. It should also be emphasized that the inclusion of additional data, such as RNA fluorescence in situ hybridization or RT-PCR, could in the future yield important information on transcriptional activity within germ cells and meiotic telomeres.

## Supplementary materials

Figure S1 RNA polymerase II (RNAPII-Ser2 and RNAPII-Ser5) distribution in spermatocytes of *N. xanthodon* at different pachytene substages, NX-02 (A–X). Pachytene spermatocytes of mole rat stained for SYCP3 (chromosome core protein; green), RNAPII-Ser5 (white), and RNAPII-Ser2 (red), showing different transcriptional regions. DNA/chromatin was stained with DAPI (cyan). Abbreviations: X: female sex chromosome; Y: male sex chromosome; XY: sex body. Scale bar, 5 μm

Figure S2 RNA polymerase II (RNAPII-Ser2) distribution in spermatocytes of *N. xanthodon* at prophase I stages (late leptotene/early zygotene, pachytene), NX-02 (A–D). Mole rat spermatocytes stained for SYCP3 (chromosome core protein; green); RNAPII-Ser2 (red), showing active transcriptional regions. DNA/chromatin was stained with DAPI (blue). Abbreviations: XY: sex body. Scale bar, 5 μm.

Figure S3 RNA polymerase II (RNAPII-Ser2) distribution in leptotene spermatocytes of *N. xanthodon*, NX-02 (A–C). Mole rat spermatocytes stained for SYCP3 (chromosome core protein; green); RNAPII-Ser2 (red), showing active transcriptional regions. DNA/chromatin was stained with DAPI (blue). Scale bar, 5 μm.

Figure S4 RNA polymerase II (RNAPII-Ser2) distribution in spermatocytes of *N. xanthodon* at prophase I stages (mid zygotene, early and mid pachytene), NX-02 (A–D). Mole rat spermatocytes stained for SYCP3 (chromosome core protein; green); RNAPII-Ser2 (red), showing active transcriptional regions. DNA/chromatin was stained with DAPI (blue). Abbreviations: X: female sex chromosome; Y: male sex chromosome; Scale bar, 5 μm.
