# Supplementary figures and images for: Unusual RNA polymerase II patterns in meiotic prophase I in Nannospalax xanthodon: insights into chromatin, telomeres, and sex chromosomes
Source: Turk J Biol. 2026 Feb 2;50(2):109–21. doi: 10.55730/1300-0152.2794 (PMC13124149; doi:10.55730/1300-0152.2794)

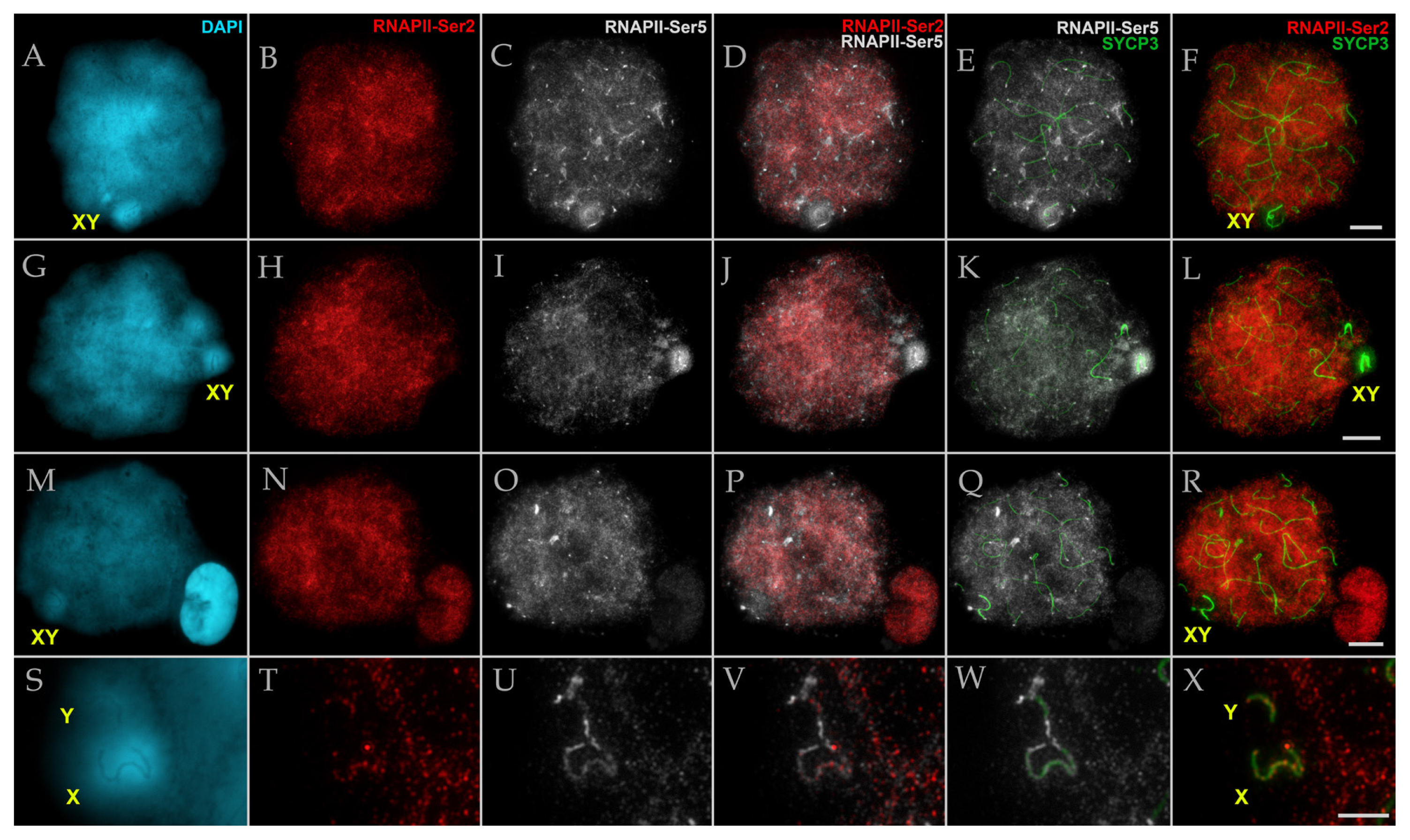

Supplement: Figure S1 — RNA polymerase II (RNAPII-Ser2 and RNAPII-Ser5) distribution in spermatocytes of N. xanthodon at different pachytene substages, NX-02 (A–X). Pachytene spermatocytes of mole rat stained for SYCP3 (chromosome core protein; green), RNAPII-Ser5 (white), and RNAPII-Ser2 (red), showing different transcriptional regions. DNA/chromatin was stained with DAPI (cyan). Abbreviations: X: female sex chromosome; Y: male sex chromosome; XY: sex body. Scale bar, 5 μm [file tjb-50-02-109s1.tif]

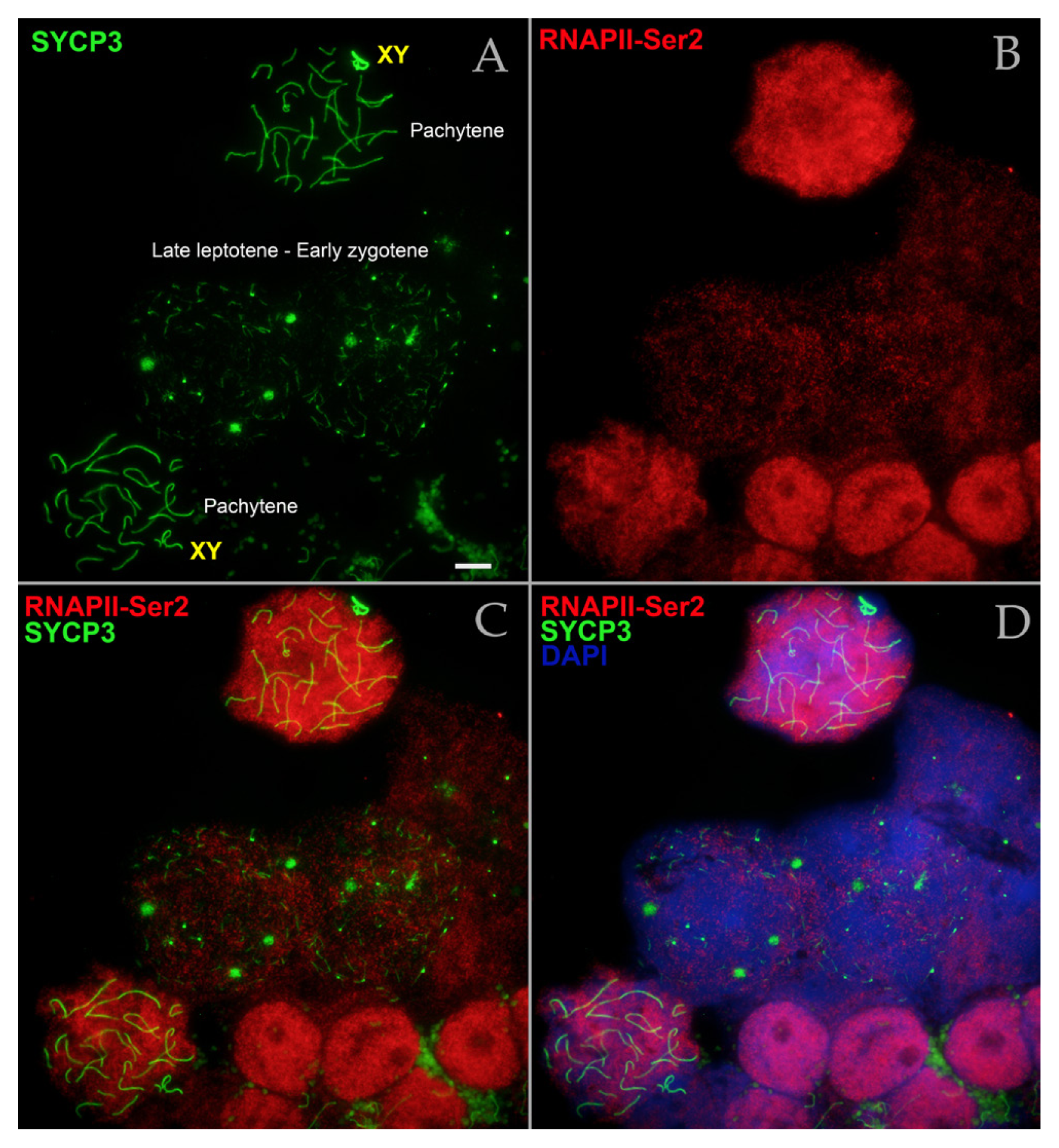

Supplement: Figure S2 — RNA polymerase II (RNAPII-Ser2) distribution in spermatocytes of N. xanthodon at prophase I stages (late leptotene/early zygotene, pachytene), NX-02 (A–D). Mole rat spermatocytes stained for SYCP3 (chromosome core protein; green); RNAPII-Ser2 (red), showing active transcriptional regions. DNA/chromatin was stained with DAPI (blue). Abbreviations: XY: sex body. Scale bar, 5 μm. [file tjb-50-02-109s2.tif]

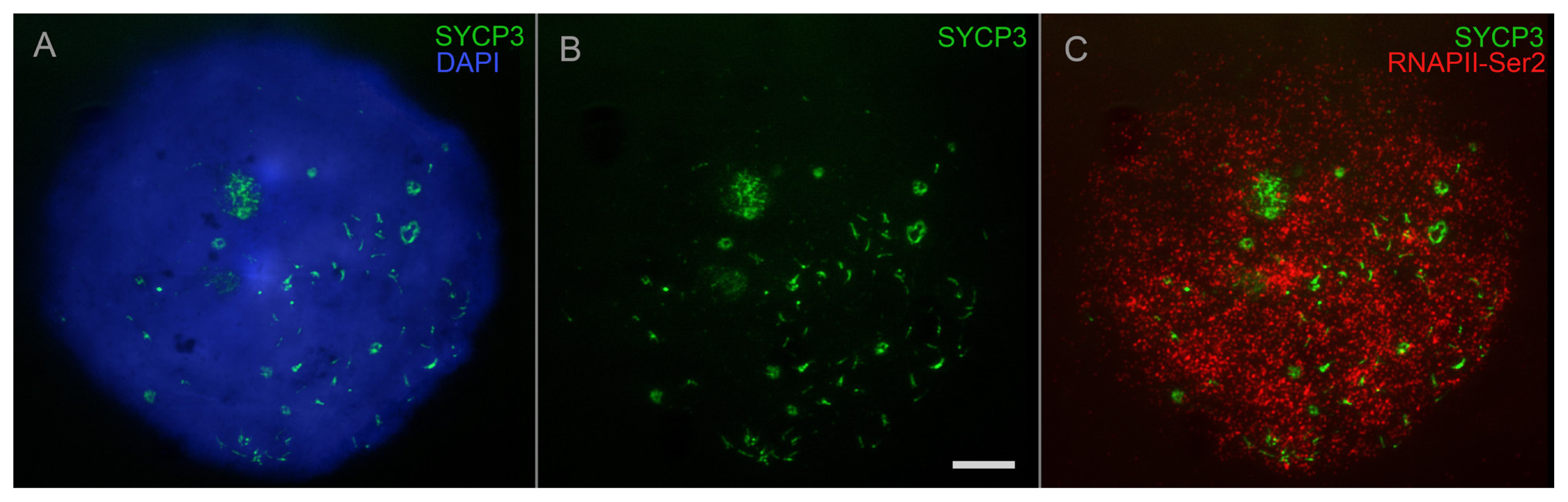

Supplement: Figure S3 — RNA polymerase II (RNAPII-Ser2) distribution in leptotene spermatocytes of N. xanthodon, NX-02 (A–C). Mole rat spermatocytes stained for SYCP3 (chromosome core protein; green); RNAPII-Ser2 (red), showing active transcriptional regions. DNA/chromatin was stained with DAPI (blue). Scale bar, 5 μm. [file tjb-50-02-109s3.tif]

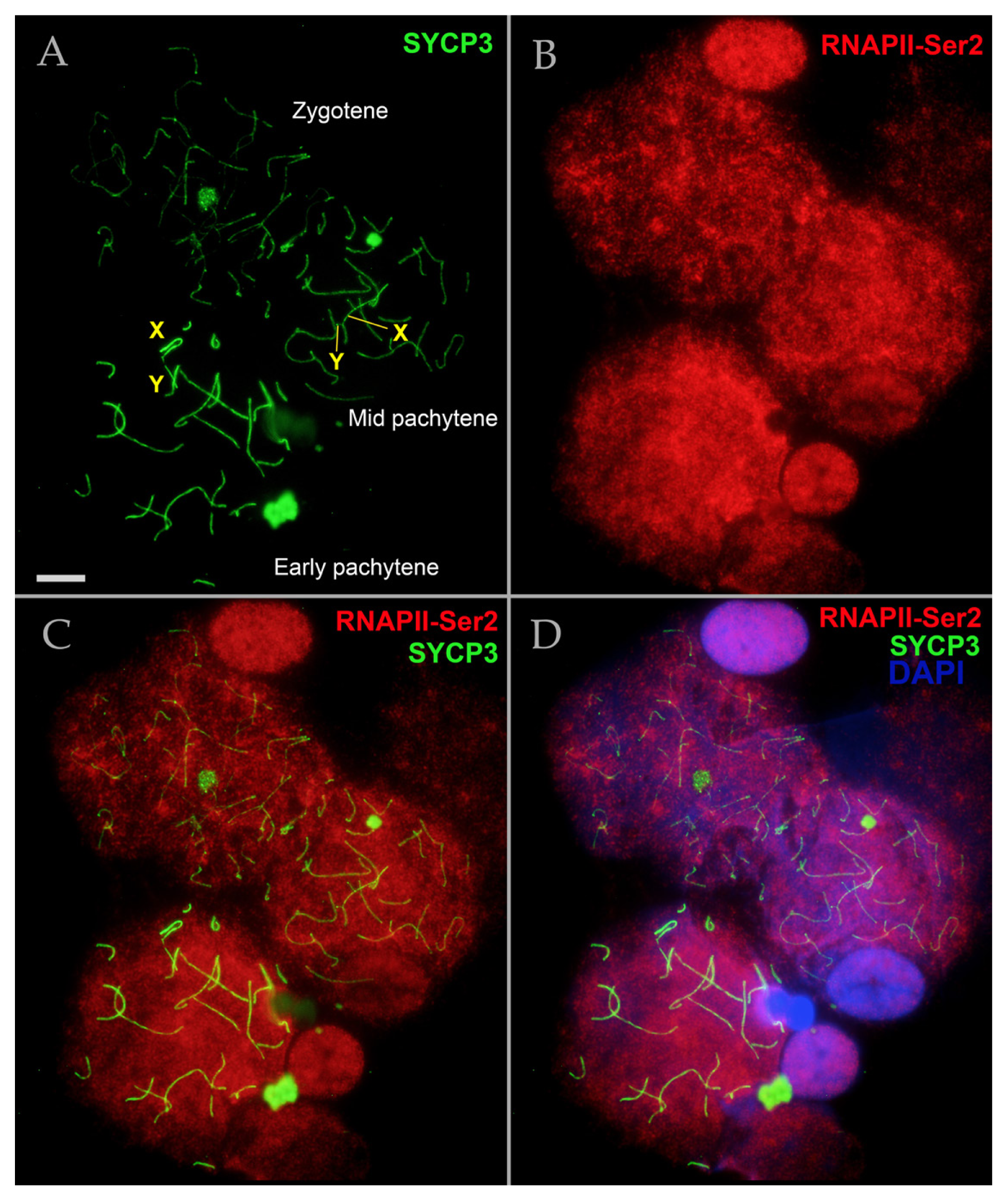

Supplement: Figure S4 — RNA polymerase II (RNAPII-Ser2) distribution in spermatocytes of N. xanthodon at prophase I stages (mid zygotene, early and mid pachytene), NX-02 (A–D). Mole rat spermatocytes stained for SYCP3 (chromosome core protein; green); RNAPII-Ser2 (red), showing active transcriptional regions. DNA/chromatin was stained with DAPI (blue). Abbreviations: X: female sex chromosome; Y: male sex chromosome; Scale bar, 5 μm. [file tjb-50-02-109s4.tif]
